# Comparison and Functional Genetic Analysis of Striatal Protein Expression Among Diverse Inbred Mouse Strains

**DOI:** 10.3389/fnmol.2019.00128

**Published:** 2019-05-24

**Authors:** Cory Parks, Francesco Giorgianni, Byron C. Jones, Sarka Beranova-Giorgianni, Bob M. Moore II, Megan K. Mulligan

**Affiliations:** ^1^Department of Genetics, Genomics and Informatics, College of Medicine, University of Tennessee Health Science Center (UTHSC), Memphis, TN, United States; ^2^Department of Pharmaceutical Sciences, College of Pharmacy, University of Tennessee Health Science Center (UTHSC), Memphis, TN, United States

**Keywords:** C57BL/6J, DBA/2J, striatum, synaptosome, proteomics, systems genetics, mass spectrometry, THC

## Abstract

C57BL/6J (B6) and DBA/2J (D2) inbred mouse strains are highly variable genetically and differ in a large number of behavioral traits related to striatal function, including depression, anxiety, stress response, and response to drugs of abuse. The genetic basis of these phenotypic differences are, however, unknown. Here, we present a comparison of the striatal proteome between B6 and D2 and relate differences at the protein level to strain differences at the mRNA level. We also leverage a recombinant inbred BXD population derived from B6 and D2 strains to investigate the role of genetic variation on the regulation of mRNA and protein levels. Finally, we test the hypothesis that differential protein expression contributes to differential behavioral responses between the B6 and D2 strain. We detected the expression of over 2,500 proteins in membrane-enriched protein fractions from B6 and D2 striatum. Of these, 160 proteins demonstrated significant differential expression between B6 and D2 strains at a 10% false discovery level, including COMT, GABRA2, and cannabinoid receptor 1 (CNR1)—key proteins involved in synaptic transmission and behavioral response. Similar to previous reports, there was little overlap between protein and transcript levels (25%). However, the overlap was greater (51%) for proteins demonstrating genetic regulation of cognate gene expression. We also found that striatal proteins with significantly higher or lower relative expression in B6 and D2 were enriched for dopaminergic and glutamatergic synapses and processes involved in synaptic plasticity [e.g., long-term potentiation (LTP) and long-term depression (LTD)]. Finally, we validated higher expression of CNR1 in B6 striatum and demonstrated greater sensitivity of this strain to the locomotor inhibiting effects of the CNR1 agonist, Δ9-tetrahydrocannabinol (THC). Our study is the first comparison of differences in striatal proteins between the B6 and D2 strains and suggests that alterations in the striatal proteome may underlie strain differences in related behaviors, such as drug response. Furthermore, we propose that genetic variants that impact transcript levels are more likely to also exhibit differential expression at the protein level.

## Introduction

High throughput and low-cost global quantification of proteins is not yet feasible. In contrast, advances in next generation sequencing technologies have resulted in the ability to identify nearly all sequence variants segregating across genomes and to profile the abundance of nearly all species of transcribed RNA present in a sample at high throughput and low cost. Quantification of relative protein abundance is a vital, albeit often absent, data level that is required to understand the flow of information from DNA sequence to complex behavior. It is well established that transcript level is not a good proxy for protein level (Gygi et al., 1999; Gry et al., 2009; Maier et al., 2009; Ghazalpour et al., 2011) and that most mRNA and protein products originating from the same cognate gene do not display a simple linear relationship (Maier et al., 2009). Therefore, to better understand biological systems and the relationship between genetic variation and behavioral variation, it is essential to identify and quantify all intervening molecular products. However, the gap between technological advancements in global protein and nucleic acid quantification has resulted in an imbalance among molecular data sets. Many data sets identifying differential transcript expression among genetically divergent individuals have been generated over the past few decades. Some of these data sets also explore the role of genetic variation in regulating transcript expression. However, there are comparatively few data sets that compare protein expression among genetically diverse individuals and even fewer that explore differential mRNA and protein expression. The paucity of proteomic data sets represents a severe knowledge gap that limits our understanding of biological systems.

Here, we compare expression of striatal proteins between the highly genetically polymorphic C57BL/6J (B6) and DBA/2J (D2) inbred mouse strains. These strains display divergence in striatal-related phenotypes, including basal striatal function (Siciliano et al., 2017), impulsivity (Peña-Oliver et al., 2016), response to novelty and stress (Cabib et al., 1998, 2002; Ventura et al., 2002), and response to haloperidol, alcohol, and drugs of abuse (Dains et al., 1996; Boone et al., 1997; Petruzzi et al., 1997; Jones et al., 1999; Kozell et al., 2005; Kapasova and Szumlinski, 2008; Tapocik et al., 2009; Siciliano et al., 2017). Striatal transcriptional profiling has been performed on these strains, including deep RNA sequencing (Bottomly et al., 2011). Profiling of the mouse striatal proteome has been performed recently within a single strain (Sharma et al., 2015), between genetically engineered mouse lines (Langfelder et al., 2016), and in the same strain following perturbation (Uys et al., 2016). A recent study by Loos et al. (2016) explored variation in the striatal synaptic proteome between B6 and seven additional strains of mice (NOD/LtJ, A/J, 129S1/SvImJ, FVB/NJ, WSB/EiJ, PWK/PhJ, and CAST/EiJ). Our study extends these findings through comparison of striatal protein levels between the highly divergent B6 and D2 strains. To better understand the relationship between genetic variation and striatal transcript and protein level, we also compare differentially expressed (DE) proteins and transcripts among the B6 and D2 strains and identify potential genetic modulation using a genetically diverse recombinant inbred BXD population derived from the B6 and D2 strain (Peirce et al., 2004; Andreux et al., 2012).

In this study, we report both novel and previously identified differences in striatal protein levels between the B6 and D2 strain. Similar to previous reports (Ghazalpour et al., 2011; Loos et al., 2016) few DE proteins demonstrated corresponding changes at the mRNA level and the majority of the strain differences detected at the protein level were not detected at the mRNA level. However, our systems genetics analysis among the BXD population revealed that gene variants that produce variation at the mRNA level appear more likely to demonstrate a corresponding change at the protein level. In contrast, the mechanisms underlying the vast majority of DE protein expression between strains remain elusive and highlight the need to better explore the proteome. DE proteins identified in our analysis may exert a functional impact on striatal function and behavior and account for some of the phenotypic variation between the B6 and D2 strains. As an example, we demonstrate a potential relationship between levels of the cannabinoid receptor 1 (CNR1), in B6 and D2 striatum and response to cannabinoids.

## Materials and Methods

### Mice Used for Quantification of Proteins in Striatum

Subjects included four male B6 and D2 mice bred in-house for two or three generations from stocks originally obtained from the Jackson Laboratory (Stock Numbers 000664 and 000671). B6 mice were 137 days-of-age (four mice) and D2 mice were 83, 102 (two mice), and 194 days-of-age at the time of harvest. All mice were maintained in same-sex group housing following weaning. Selected mice are in the age range of normal adulthood (3–6 months) and were naïve to any testing. Mice were fed a standard laboratory diet (Harlan Teklad 7912) with food and water available *ad libitum* and maintained on a 12 h light/dark cycle (lights on at 06:00 h and off at 18:00 h). These procedures were approved by the UTHSC Institutional Animal Care and Use Committee (protocol 16-077).

### Dorsal and Ventral Striatum Dissection

Male mice were euthanized between 14:00 h and 17:00 h using isoflurane followed by decapitation. The brain was removed and striatum was sub-dissected from fresh tissue using the following procedure. Whole brain was placed dorsal side down in a mouse brain matrix. Two razor blades were then placed in the matrix to retrieve the striatal brain section. The first blade was positioned 3 mm posterior to the olfactory bulb and the second blade was positioned 2 mm posterior to the first blade and just rostral to the optic chiasm. The entire dorsal and ventral striatum from each hemisphere were sub-dissected from the slice using visual landmarks and placed in a tube. Dissections were carried out with the brain matrix and a nonstick plastic dissection mat placed on a temperature monitored cooling core (Biocision XT Starter CoolBox) pre-chilled at −20°C. Each hemisphere was immediately flash frozen in liquid nitrogen and stored at −80°C.

### Isolation of Membrane and Mitochondrial Enriched Protein Fractions

Isolation of crude membrane and soluble fractions was performed as described previously (Distler et al., 2014b). All steps were performed at 4°C. Briefly, the striatum was mechanically homogenized with a motorized tissue grinder in lysis buffer [5 mM HEPES, 0.32 M sucrose, 1× protease and phosphatase inhibitor cocktail (Thermo Fisher), pH 7.5] at a weight:volume ratio of 1:10 (1 mg per 10 μL). Homogenates were centrifuged at 1,000× *g* for 10 min. Following removal of the pellet (P1), the supernatants were collected and centrifuged again at 12,000× *g* for 10 min. The soluble protein fraction was collected from the supernatant, and the resulting pellet (P2) was collected as the crude membrane and mitochondrial enriched protein fraction and resuspended in PBS. Fractions were stored at −80°C prior to processing for proteomics analysis. Protein levels in extracts were quantified using a BSA Assay Kit (Thermo Scientific) on the Agilent Nanodrop device. Note that the P2 was retrieved from each individual mouse and samples were not pooled for the proteomics analysis described below.

### Proteomics Analysis

Sample preparation was performed using the iST Sample Preparation Kit (PreOmics GmbH; Kulak et al., 2014) exactly as described, except that protein samples were evaporated in a speed vacuum centrifuge to dryness, and a custom storage solution (98% H_2_O, 2% ACN, 0.1% TFA) was used instead of the supplied LC-Load solution to dissolve the peptide samples after drying. The peptide solutions were mixed in a 1:1 (v:v) ratio with a 25 fmol/μL solution of yeast alcohol dehydrogenase 1 (ADH1, P00330) tryptic digest standard (Waters Corporation). All samples were analyzed on a Synapt G2-Si quadrupole time-of-flight (QTOF) tandem mass spectrometer with ion mobility separation (IMS; Waters Corporation). The Synapt G2-Si was interfaced to an Acquity UPLC M-Class nano-LC system (Waters Corporation). The LC-MS/MS system was controlled *via* the MassLynx software suite (v. 4.1). Samples (2 μL) were applied to an on-line LC pre-column trap column (Symmetry C18, 100 Å, 5 μm, 180 μm × 20 mm, Waters Corporation) by a partial loop injection with the aid of the autosampler component of the Acquity M-class system. Peptides were separated on a nano flow UPLC C18 column (HSS T3, 1.8 μm, 75 μm × 250 mm, Waters Corporation) with a 180 min linear gradient of 2%–40% mobile phase B (ACN/0.1% formic acid) and mobile phase A (H_2_O/0.1% formic acid) at a flow rate of 300 nL/min. The analytical column temperature was kept at 45°C. The eluted peptides were introduced into the mass spectrometer on-line *via* a nano electrospray source with temperature set at 80°C and capillary voltage of 2.8 kV. For lock-mass correction, [Glu^1^]-fibrinopeptide B standard was infused through the fluidics system of the instrument. The data were acquired in an HDMS^E^ data-independent acquisition mode (Distler et al., 2014a). The instrument quadrupole was set to transmit ions with m/z >300. The mass spectrometer acquisition parameters were set into an HDMS^E^ method created through MassLynx. The method included settings for the TOF to detect ions in the 50–2,000 m/z range, and for IMS that preceded peptide precursor ion fragmentation in the transfer cell. The TOF was operated in resolution mode (resolution >20,000 FWHM). The low collision energy was set at 6 V and the high collision energy was set to be drift time-specific and ramped during acquisition from 17 V to 60 V. This setting was included in the HDMS^E^ method and implemented with the aid of a look-up table created in MassLynx (Silva et al., 2006a). The LC-MS/MS data were analyzed with the Progenesis QI for proteomics (v. 2.1, Nonlinear Dynamics) software platform. The software default peak-picking settings were used to process the raw data. Peptides were identified *via* the PLGS search engine (v. 3.0.3, Waters) from searching a non-redundant mouse database, which contained *ca* 20,000 protein entries. The search allowed for a maximum of one trypsin missed peptide cleavage, static modification of cysteine (carbamidomethylation), and variable modification of methionine (oxidation). The peptide false discovery rate was set to less than 4%. Identified proteins were displayed according to the protein grouping method and were quantified with the “Hi-3” method (Silva et al., 2006b) using ADH1 as an internal calibrant for absolute quantification. The normalized protein abundance fold changes and related statistical significance among groups were calculated in Progenesis using the software default settings. All 2,591 detected proteins are summarized in Supplementary Table S1. Proteins were considered differentially expressed (DE) based on a false discovery level (*q*-value) of 10%.

### Missense SNP Analysis

The reference mouse database used for peptide identification is based upon B6 sequence; therefore, missense mutations between B6 and D2 have the potential to impact detection and/or expression. No mismatches are allowed during peptide identification in our method, thus missense mutations may have an impact on detection, especially detection of D2 peptides. However, the impact of missense mutations on expression is mitigated by the fact that the three most abundantly detected peptides are used for protein quantification in our analysis. Missense mutations that alter protein function can also have an indirect impact on expression level independent of technical issues due to the adaptation of redundant and scale-free biological systems. Previously, Lenselink et al. (2015) identified 27 proteins (STXBP1, ATP2B2, BHLHE41, BLM, GDI1, LGI2, SIGLEC1, AURKAIP1, BABAM1, C1QB, COPS2, DMWD, FAM214A, GPNMB, IGSF8, ISG20L2, KBTBD4, MAP1S, MBLAC1, ORC6, PVRL1, REST, ROBO4, SLC12A5, SSPN, USP8, ZC3H6) containing tryptic peptides with missense mutations between B6 and D2. Of the 27 affected proteins, we detected STXBP1, GPNMB, COPS2, ATP2B2, GDI1 in our preparations. Only STXBP1 demonstrated evidence of differential expression (B6_avg_ = 8.43 ± 0.97 fmol, D2_avg_ = 5.88 ± 0.44, *q* = 0.16). Given the high level of expression detected for this gene in both strains (data set average expression = 2.8 fmol), we did not remove any proteins from the analysis due to overlapping missense mutations.

### Enrichment Analysis

**Figure 1:**
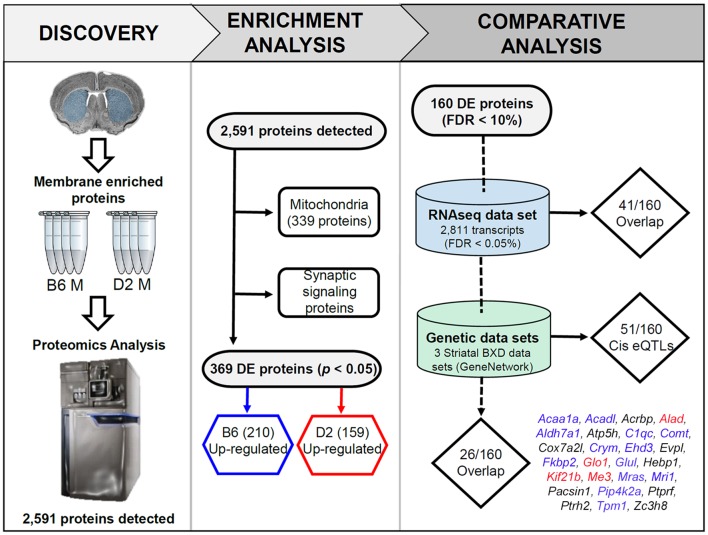
Overview of the discovery, enrichment, and comparative analyses. In the discovery phase, membrane and mitochondrial enriched protein fractions were collected from the striatum (dorsal and ventral) of four males from the B6 and D2 inbred mouse strains. For the enrichment analysis, all detected proteins were first queried for enrichment of functional terms using the ENRICHR web service. This resulted in detection of 339 mitochondrial genes. The remaining proteins were found to be enriched for synaptic signaling pathways (summarized in Figure 2). In the last phase of enrichment analysis, 369 differentially expressed (DE) proteins were divided into two groups for additional enrichment analysis based on higher expression in the B6 or D2 strain. The results of this analysis can be found in Figure 3. For comparative and genetic analysis the top 160 DE proteins based on a 10% FDR were compared against two related data sets. The first data set consisted of striatal gene expression profiled in male B6 and D2 mice by RNA-seq. The second data set included expression quantitative trait loci (eQTL) mapping in three different striatal expression data sets from a recombinant inbred progeny—the BXD population—derived from the B6 and D2 strains. The final list of 26 proteins with differential mRNA expression and evidence of genetic regulation of gene expression in the BXD panel is shown above (blue, red, and black text indicates higher expression of the B6 allele, D2 allele, or a mixture of both across the three data sets, respectively). A summary of the overlap analysis can be found in Supplementary Table S3.

An overview of the experimental pipeline, including the enrichment analysis, can be found in Figure 1. Two different enrichment analyses were performed using tools available at the Enrichr (Kuleshov et al., 2016) website^1^. In the first analysis, enrichment was assessed for all detected proteins. In the second exploratory analysis, the threshold for DE proteins was relaxed to a nominal level (*p* < 0.05). Two gene sets were created from this list corresponding to proteins with higher relative expression in either B6 or D2. For both analyses, official gene symbols for detected proteins were entered into the query page and enrichment of functional terms was explored using the KEGG 2016 database. Default settings were used for Enrichr analysis. An adjusted *p*-value < 0.05 was used as the criterion of significance. The *p*-value was computed for each term from the Fisher exact test. The adjusted *p*-value was calculated by running the Fisher exact test many times on random data sets to compute a mean rank followed by calculation of the deviation of each term from this rank.

### Comparison With Striatal RNA-seq Dataset

An overview of the experimental pipeline, including the comparison between proteomics and RNA-seq data, can be found in Figure 1. We compared our results with a deep RNA-seq data set generated for 21 striatal samples (11 D2 and 10 B6 males) at a short read (~50 bases) depth of ~22 million reads per sample using the Illumina GAIIx platform (Bottomly et al., 2011). The data set (Supplementary Table S1 in the original manuscript) was filtered from 16,183 detected transcripts to 2,811 based on a 5% false discovery rate. Updated gene symbols were obtained for the 2,811 DE transcripts using the associated Ensembl gene identifiers and tools available at Biomart^2^. Gene symbol was then used to match across data sets (2,811 DE transcripts vs. 160 DE proteins) and the direction of the strain effect (higher relative expression in B6 or D2) was compared.

### Functional Genetic Analysis

An overview of the experimental pipeline, including the integration of BXD genomic data, can be found in Figure 1. We queried genomic resources available at GeneNetwork (GN^3^). Specifically, we looked for evidence of genome-wide significant or suggestive cis-modulation of striatal mRNA expression for DE proteins among recombinant inbred BXD strains derived by crossing and inbreeding B6 and D2 strains. We reasoned that in some cases alterations at the protein level would also appear at the mRNA level, especially if caused by the presence of a functional genetic variant. Three striatal microarray expression data sets were used in the analysis. The Illumina data set consisted of striatal gene expression profiles measured across 54 BXD strains using the Illumina mouse-6 v1.1 expression beadchip array [HQF BXD Striatum ILM6.1 (Dec10v2) RankInv, GN285]. The M430 data set consisted of striatal gene expression profiles measured across 29 BXD strains using the Affymetrix Mouse Genome 430 2.0 array [HBP Rosen Striatum M430V2 (Apr05) RMA Clean, GN69]. The Exon data set consisted of striatal gene expression profiles measured across 29 BXD strains using the Affymetrix Mouse Exon 1.0 ST Array [HQF Striatum Affy Mouse Exon 1.0ST Gene Level (Dec09) RMA, GN399]. Each data set was generated on different platforms that featured different probe types and normalization/summary methods. For all data sets, gene expression was averaged for strain replicates (typically consisting of a single male and female animal) and reported as the average log_2_ expression for each probe or probe set. In GN, each data set is normalized to have an average log_2_ expression of 8 with a standard deviation of ±2 units. Traditional interval mapping in GN to identify cis expression quantitative trait loci (cis eQTLs) was performed using a simple regression method (Haley-Knott or HK) to compute QTL probability given strain genotypes and probe/probe set expression averaged by strain (Chesler et al., 2005; Mulligan et al., 2017). For traditional interval mapping, genome-wide suggestive (adjusted *p* < 0.63) and significant (adjusted *p* < 0.05) thresholds were determined based on 1,000 permutations of the trait data for each expression trait (GN default). An eQTL was considered to be cis-modulated if the location of the protein-coding gene fell within the 95% confidence interval (e.g., the right and left chromosomal position at which the LOD score dropped 1.5 units from the LOD score of the peak marker). For each data set, all non-specific probes/probe sets and probes/probe sets overlapping a sequence variant that had an obvious impact on gene expression measures were excluded. This included Illumina probes ILM6960092, ILM102230086, ILM6290242, ILM5860632, ILM3440717, representing the metabotropic glutamate receptor (*Grm4*), hemoglobin beta 2 subunit (*Hbb-b2*), proteasome beta subunit type-5 (*Psmb5*), mitochondrial pentatricopeptide repeat domain-containing protein 3 (*Ptcd3*), and sodium/calcium exchanger 1 (*Slc8a1*), respectively. All of these probe sets overlapped one or more sequence variants and higher expression was associated with inheritance of the B allele in the BXD population. This finding could be due to the interference of the overlapping variant on probe hybridization or a true cis eQTL. Since the impact of the overlapping variant on gene expression measurements could not be determined, these probes were excluded from analysis. Suggestive or significant eQTLs detected in two or three different microarray expression platforms were considered to be especially robust. Significant genes and eQTLs were visualized using the PhenoGram visualization tool^4^.

### Western Blot Analysis

From remaining crude membrane preps (three per strain), 20–30 μg of protein was loaded into a pre-cast SDS Page gel and run at 180 V for 1 h. Proteins were then transferred to a nitrocellulose membrane with a wet transfer at 30 V for 1 h. The membrane was then stained using the Swift Stain kit (GBioscience) to quantify total protein on membrane. The membrane was then imaged, destained, and put in blocking solution, 5% Milk (Biorad) in TBST (1× TBS + 0.05% Tween), for 1 h. The membrane was then probed (1:500 dilution) with the primary antibody (Abcam anti-CNR 1 rabbit antibody, ab107817, rabbit) overnight at 4°C. The next morning the membrane was washed, probed (1:5,000 dilution) with the secondary antibody (Invitrogen anti-rabbit goat antibody 31466) for 1 h at room temperature, and washed a final time. Protein signal was then detected using the Novex™ ECL Chemiluminescent Substrate Reagent Kit (Invitrogen) and images were acquired using an Amersham Imager 600 (GE). Image analysis was performed using the software program imageJ using the grayscale method. For both images acquired through chemiluminescence and Swift Staining, mean grayscale values for protein bands and background were obtained, and background values were subtracted by protein band values. The resulting corrected protein band values acquired from chemiluminescent detection in each sample were then normalized using the corresponding Swift Stain corrected protein band level (original blot shown in Supplementary Figure S1).

Two additional westerns were run using different individual B6 and D2 male mice and different protein preparation methods. To reproduce strain differences in total protein extracts one B6 and D2 mouse (aged 141 and 165 days, respectively) were used to generate total protein extracts from whole brain as described previously (Mulligan et al., 2019). Protein quantification, loading and transfer, and membrane probing occurred as described above with the exception that 40 μg of protein was loaded per lane and the membranes were not stained using Swift Stain. Instead, the membrane was cut into two sections corresponding to a molecular weight of 50 or 37 kDa. The 50 kDa section was probed with anti-CNR1 as described above and the 37 kDa section was probed (1:5,000 dilution) with primary antibody (Cell Signaling anti-GAPDH antibody, 31466, rabbit) using the same incubation and wash schedule described above. Both membranes were probed with secondary antibody and protein signal was detected as described above with the exception that GAPDH levels were used for normalization. Original blot shown in Supplementary Figure S2. To reproduce strain differences using the same membrane enrichment method, the striatum was dissected from two different B6 and D2 mice aged 167 days and membrane enriched and soluble fractions were collected as described above. Western analysis was also performed as described above with the exception that the membrane was divided into three pieces corresponding to molecular weights of 250–75 kDA, 50 kDa, and 37 kDa. The large molecular weight membrane was stained with the Swift Stain kit and the 50 kDa and 37 kDa membranes were probed with antibodies for CNR1 and GAPDH, respectively, as described. CNR1 levels were normalized using protein band intensities from the Swift stained membrane. Original blots shown in Supplementary Figure S2.

### Functional Characterization of Motor Response to the CNR1 Agonist Δ9-Tetrahydrocannabinol (THC)

Male B6 and D2 mice (eight per strain treated with THC and two per strain treated with vehicle) were purchased from the Jackson Laboratory. All mice were aged to at least 60 days prior to testing. At least 1 week prior to testing, animals were separated into individual housing and handled daily. Handling consisted of lifting mice in either cupped hands (avoiding any lifting by the tail) or a plastic lid from a pipette tip box. Food and water were provided *ad libitum* and mice were maintained on a 12 h:12 h light:dark cycle. All testing was performed during the light cycle from 7 am to 4 pm. All animal activities were approved by the University of Tennessee Health Science Center Institutional Animal Care and Use Committee.

THC was formulated in an ethanol:cremophor:saline (5:5:90) vehicle followed by filter sterilization. The resulting formulation was stored in the dark and under refrigeration (4°C) in a septum-sealed vial. Vehicle (VEH) was prepared in the same manner. THC and VEH were administered by intraperitoneal (i.p.) injection at a dose of 10 mg/kg such that a 30 g mouse received a 100 μL injection.

Two motor phenotyping tests, the Ring Test and the Open Field Test, were used to measure THC response. The Ring Test was adapted from Pertwee (1972) and used to assess sensitivity to THC. The amount of time spent immobile on a horizontal wire ring was measured over 5 min. The horizontal ring (equivalent to a chemistry ring clamp) was approximately 5.5 cm (2.2 inch) in diameter and positioned 16 cm (6.5 inch) from the ground on a ring stand. Horizontal bars holding rings were spaced ~8 inches apart such that up to four mice could be placed on the ring apparatus at the same time. Mice were gently placed across the ring and observed for 5 min. Mice that climbed down the apparatus were gently replaced up to three times. Immobility (no movement of any kind for at least 3 s was scored using AnyMaze (Stoelting) software. Finally, motor activity in response to THC was measured as total distance traveled during a 10 min period in a 40 × 40 × 40 cm open field. Activity was analyzed using AnyMaze software.

On day 0, all mice were injected with VEH. At 30 min post-injection, mice were placed on the horizontal ring for 5 min. At 75 min post-injection, mice were placed in the center of the open field for 10 min. On days 1 through 3 mice were given a VEH (*n* = 2 per strain) or THC (*n* = 8 per strain) injection. Immobility was assessed 30 min later on day 1 and day 3. Motor activity was assessed 75 min later on both days. Multiple factor ANOVA was used to identify strain and condition (THC or VEH) main effects and interactions.

## Results

### Divergence in the Expression of Striatal Proteins Between the B6 and D2 Strains

**Figure 2:**
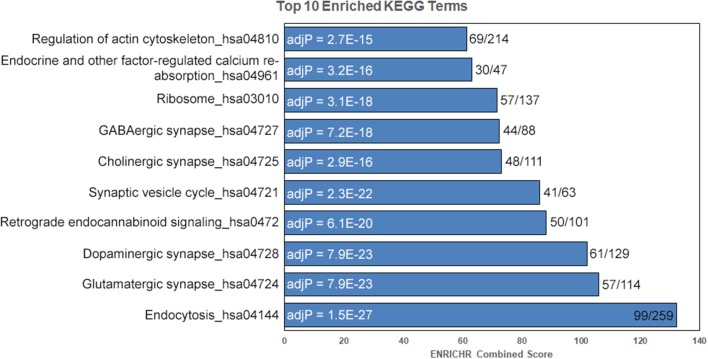
Enrichment for key striatal signaling pathways. Following removal of mitochondrial proteins, enrichment analysis was performed on detected proteins using the ENRICHR web service. Enriched KEGG pathway terms are shown to the left and adjusted *P*-values (adjP) following correction for multiple testing are shown in the bars. The ratio of terms in our data set compared to the total number of KEGG terms is shown to the left of the bars. Terms sorted by ENRICHR Combined Score.

We detected 2,591 proteins in membrane and mitochondrial enriched preparations from B6 and D2 striatum (Figure 1, Supplementary Table S1). Of these proteins, 339 were associated with mitochondria. The remaining detected proteins were significantly enriched for the following KEGG (Kyoto Encyclopedia of Genes and Genomes) pathway terms: endocytosis, glutamatergic synapse, dopaminergic synapse, retrograde endocannabinoid signaling, synaptic vesicle cycle, cholinergic synapse, GABAergic synapse, ribosome, endocrine and other factor-regulated calcium reabsorption, and regulation of the actin cytoskeleton (Figure 2).

**Figure 3:**
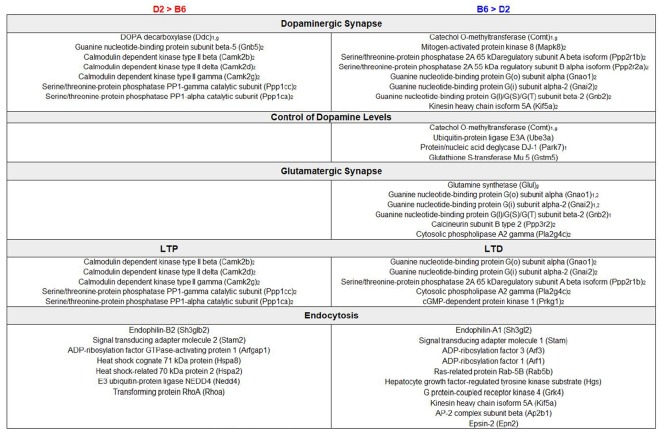
Enrichment analysis of DE proteins. Summary of DE genes by functional categories related to synaptic signaling pathways is shown to the left for higher relative expression in D2 and to the right for higher relative expression in B6. Subscript text represents presynaptic (1), postsynaptic (2), or glial (g) expression. Functional analysis of DE proteins suggests differences in dopaminergic and glutamatergic signaling with a potential impact on learning and behavior.

We performed an exploratory analysis into the functional consequences of differential protein expression using a nominal *p*-value threshold (*p* < 0.05) to select DE proteins (Figure 1). We then compared enriched biological terms between proteomics-derived gene sets with higher relative expression in either D2 or B6. DE proteins with higher relative expression in D2 striatum were significantly enriched for KEGG terms including metabolic pathways, dopaminergic synapse, long-term potentiation (LTP), and cAMP signaling pathway (Figure 3, Supplementary Table S2). Proteins with higher relative expression in B6 were significantly enriched for KEGG terms related to metabolic pathways, dopaminergic synapse, long-term depression (LTD), and glutamatergic synapse (Figure 3, Supplementary Table S2). In addition, proteomics-derived gene sets with higher relative expression in B6 were enriched for MGI Mammalian Phenotype terms related to abnormal activity levels, neurotransmission, pharmacokinetics, and dopamine level.

### Comparison of DE Protein and mRNA Expression

We selected 160 of the most significant DE proteins for a comparative analysis of mRNA expression based on a 10% FDR criterion (Supplementary Table S3). To better understand the relationship between baseline striatal protein and mRNA expression, we compared expression of these 160 proteins to striatal mRNA expression generated from many (≤10) B6 and D2 male mice using an RNA-seq platform (Bottomly et al., 2011). The comparative analysis revealed that only 41 of the 160 DE proteins (26%) demonstrated significant strain differences at the mRNA level. Of these 41 DE proteins, 31 (76%) demonstrated the same direction of the strain effect at both the protein and mRNA level.

### Genetic Regulation of DE Proteins

To evaluate whether genetic factors contribute to strain differences in striatal protein expression, we performed a genetic linkage analysis across three striatal microarray data sets generated from the BXD population. This population was created by crossing the B6 and D2 strains and then inbreeding the recombinant progeny to generate a large panel of genetically diverse inbred strains. This well-characterized population has been profiled for a number of traits, including striatal gene expression (see “Materials and Methods” section). Because all of the BXD strains have been densely genotyped, the pattern of inheritance of each strain is known or can be inferred, for nearly all positions in the genome. Within each BXD striatal expression data set, linkage analysis can be used to associate inheritance of the B6 or D2 parental allele with variation in expression of the cognate gene among BXD strains. The result is identification of cis eQTLs—polymorphic gene loci in which inheritance of one parental allele is associated with higher relative expression of the transcript encoded by that locus.

**Figure 4:**
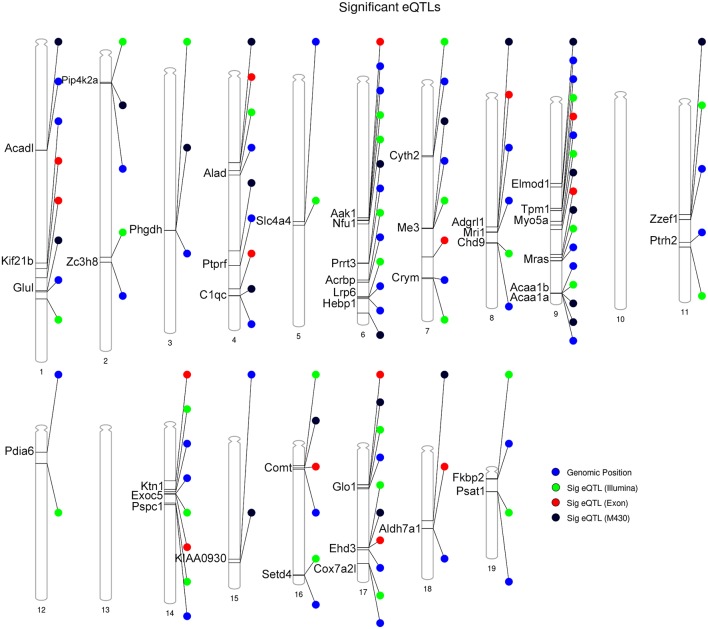
Summary of DE proteins whose transcripts demonstrate significant local modulation by a cis eQTL in the BXD family. Gene position for DE proteins indicated by the blue circle. Position of peak markers significantly associated with cognate gene expression is provided for each striatal microarray expression platform. Illumina = Illumina mouse-6 v1.1 expression beadchip array; the M430 = Affymetrix Mouse Genome 430 2.0 array; Exon = Affymetrix Mouse Exon 1.0 ST Array. Genome-wide significance of marker associations based on 1,000 permutations. Thirty genes demonstrate the same strain effect on both transcript and protein levels (e.g., higher expression associated with the B6 allele relative to the D2 allele). Thirteen genes demonstrate mismatched expression: *Acrbp*, *Adgrl1*, *Chd9*, *Cox7a2l*, *Hebp1*, *Nfu1*, *Prrt3*, *Psat1*, *Pspc1*, *Ptprf*, *Slc4a4*, *Zc3h8*, and *Zzef1*.

Following eQTL analysis, we identified 51 of 160 DE proteins (>30%) whose expression may be modulated at both the mRNA and protein level by segregating sequence variants in the cognate gene (Supplementary Table S4). The expression of 43 of the 51 DE protein-coding genes was modulated by a significant cis-eQTL in at least one striatal expression data set from the BXD population (Figure 4). Of the 51 DE proteins demonstrating significant or suggestive genetic regulation of the cognate gene, 26 (~51%) also displayed divergent expression at the mRNA level between the B6 and D2 strain in the deep RNA-seq data set (Supplementary Table S3). Several of these proteins, including ALAD, ALDH7A1, COMT, COX7A2L, and GLO1, demonstrated differential expression at the mRNA level based on the RNA-seq data set and evidence of genetic regulation of gene expression based on the BXD data sets. These genes are known to contain functional mutations that result in altered mRNA and protein levels between strains (Bishop et al., 1986; Mercer et al., 1991; Claudio et al., 1997; Bhave et al., 2006; Freeman et al., 2006; Williams et al., 2009; Kember et al., 2010; Li et al., 2010; Lapuente-Brun et al., 2013; Wang et al., 2016; Mulligan et al., 2019). In addition, GABRA2, MYO5A, and NNT are known to contain gene variants that alter protein levels (Mercer et al., 1991; Freeman et al., 2006; Mulligan et al., 2019). However, genetic regulation (without matched changes at the mRNA level in the RNA-seq data set) was only detected for MYO5A, and differential mRNA expression in the RNA-seq data set (without matched genetic regulation) was only detected for GABRA2. No difference in expression in the RNA-seq data set or genetic regulation in the BXD data sets was detected for NNT. The precise gene variants and molecular mechanisms underlying expression variation of the remaining putative genetically modulated DE proteins are not known. However, the vast majority of B6 and D2 polymorphic variants in these genes are non-coding.

### B6 Mice With Higher Levels of Synaptic CNR1 in the Striatum Are More Sensitive to the Locomotor Inhibiting Effects of THC

The majority of DE proteins detected in our proteomics analysis (109 of 160 or 68%) demonstrated differential expression at the protein level without concomitant changes at the mRNA level or evidence of direct genetic regulation of the cognate gene. These proteins are especially interesting because they may contribute to behavioral variation among strains, however, the mechanisms that lead to their differential expression are unknown. To begin to explore these enigmatic DE proteins we first selected the CNR1 for further evaluation. CNR1 was a rational first choice based on: (1) the simplicity of the receptor signaling system (CNR1 is the primary receptor in neuronal cells unlike GABA or Glutamate receptor signaling pathways); (2) the availability of highly selective pharmacological reagents; (3) the availability of specific molecular reagents (i.e., well-characterized antibodies); and (4) the availability of well-defined behavioral assays to evaluate receptor function. Expression of CNR1 is abundant in the striatum and throughout the basal ganglia where it plays an important role, along with endogenous cannabinoids, in modulating neurotransmission and motor activity. Activation of CNR1 by THC and other cannabinoid agonists produces a spectrum of behavioral responses including diminished locomotor activity, catalepsy, hypothermia, and analgesia. Genetic deletion or pharmacological inactivation of CNR1 abolishes these effects (Rinaldi-Carmona et al., 1994; Compton et al., 1996; Ledent et al., 1999; Zimmer et al., 1999; Huestis et al., 2001). Furthermore, cataleptic responses to THC are ablated in mice with selected deletion of CNR1 in striatal medium spiny projection neurons (Monory et al., 2007). Taken together, alteration of CNR1 levels or function in striatum is highly likely to specifically influence motor responses to THC. Therefore, we hypothesized that strain differences in the expression of synaptic CNR1 in striatum would correspond to differential sensitivity to the cataleptic and motor depressant effects of the CNR1 agonist THC.

**Figure 5:**
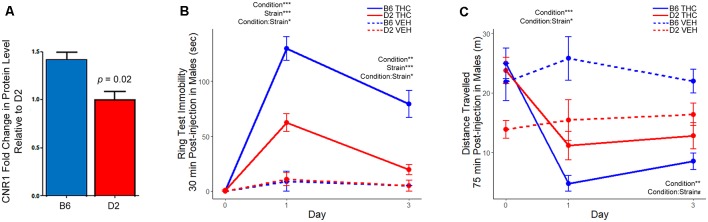
Differential strain expression of cannabinoid receptor 1 (CNR1) protein in striatum and differential strain response to Δ9-tetrahydrocannabinol (THC). **(A)** Validation of CNR1 striatal expression between B6 and D2 using traditional Western blot analysis. Higher expression in the B6 strain relative to D2 was validated in the same samples used for proteomics analysis (*n* = 3). Original membranes are shown in Supplementary Figure S1. **(B)** The THC group included eight mice per strain and the vehicle (VEH) control group included two mice per strain. Multiple factor ANOVA was used to analyze the effect of condition, strain, and interaction effects on Days 1 and 3. There was a significant strain, condition, and strain-by-condition effect of THC on mobility in the ring test on Day 1 and 3. Male B6 mice exhibited more immobility relative to the D2 strain following THC treatment. **(C)** The THC group included eight mice per strain and the VEH group included two mice per strain. Multiple factor ANOVA was used to analyze the effect of condition, strain, and interaction effects on Days 1 and 3. There was a significant condition and strain-by-condition effect of THC on activity in the open field on Day 1. On Day 3, there was a significant condition, and trend for a strain-by-condition effect of THC on activity in the open field. Male B6 mice exhibited greater suppression of motor activity in the open field relative to the D2 strain following THC treatment. Note that the D2 vehicle control group (*n* = 2) appeared to have much lower activity in the open field on Day 0. However, the study is designed such that all groups (treatment and vehicle control) also have baseline activity recorded on Day 0, mitigating the effects of the cohort effect observed in the D2 VEH group.

To test this hypothesis, we first confirmed higher expression of the CNR1 protein in B6 relative to D2 in the same samples used for proteomics by Western blot analysis (Figure 5A, Supplementary Figure S1). We also performed additional Western analyses to confirm the strain differences in CNR1 expression in three additional B6 and D2 mice in striatal membrane-enriched fractions and whole brain total extracts (Supplementary Figure S2). We then measured sensitivity to the motor depressing effects of a 10 mg/kg (*i*.*p*.) dose of THC in male B6 and D2 mice using two different assays. The first assay, the ring test, was developed by Roger Pertwee in the 1970s as a test to measure the cataleptic effects of cannabis extracts in mice (Pertwee, 1972). In the ring test, immobility on a horizontal metal ring was used as a measure of THC-induced catalepsy. Male B6 mice displayed greater immobility in the ring test 30 min following a 10 mg/kg injection of THC (Figure 5B) compared to D2. The effect of THC on immobility was also longer lasting in the B6 strain relative to the D2 strain, which had nearly returned to baseline mobility levels following three daily injections of THC. In the second assay, activity in a novel open field was used as a measure of THC-induced suppression of motor activity. B6J males showed a dramatic and significant reduction in open field activity relative to D2 males on both day 1 and day 2 of THC treatment (Figure 5C).

## Discussion

Here, we have provided the first glimpse into differential protein expression between B6 and D2 males in the striatum and performed an exploratory analysis into the causes and consequences of this variation. We detected over 2,500 proteins and observed enrichment for both mitochondrial and synaptic proteins. This was an expected result based on our crude membrane and mitochondrial enriched preparation.

Consistent with our tissue type, striatum, the top 10 enriched pathway terms following filtering of mitochondrial genes included neuronal signaling pathways (e.g., glutamatergic synapse, dopaminergic synapse, retrograde endocannabinoid signaling, synaptic vesicle cycle, cholinergic synapse, and GABAergic synapse; Figure 2). The striatum is a relatively homogenous tissue consisting of generally well-defined cell types, the majority of which are GABAergic, medium spiny neurons (Kreitzer and Malenka, 2008) that receive glutamatergic input from cortical and thalamic regions, GABAergic input from other medium spiny neurons, and dopaminergic input from the ventral tegmental area and substantia nigra. These findings validate our unbiased proteomics approach and demonstrate that we are able to detect a wide range of pre- and post-synaptic proteins using crude membrane enriched protein preparations.

Comparison of the striatal proteome between male B6 and D2 resulted in the identification of 160 DE proteins. Among these were eight proteins—ALAD, ALDH7A1, COMT, COX7A2L, GABRA2, GLO1, MYO5A, NNT—containing known functional sequence variants impacting protein level between B6 and D2 (Bishop et al., 1986; Mercer et al., 1991; Claudio et al., 1997; Bhave et al., 2006; Freeman et al., 2006; Williams et al., 2009; Kember et al., 2010; Li et al., 2010; Lapuente-Brun et al., 2013; Wang et al., 2016; Mulligan et al., 2019; Supplementary Table S3). Identification and replication of these previously identified moderate effect strain differences in protein expression is a further validation of our approach. Increasing the number of biological replicates in future studies is expected to increase detection of smaller protein expression differences between strains. Of note, six of the eight genes (*Alad*, *Aldh7a1*, *Comt*, *Cox7a2l*, *Gabra2*, *Glo1*) containing strong variants that alter protein levels also demonstrate significant alterations in mRNA level (based on comparison with a deep RNA-seq data set, Supplementary Table S3) consistent with the changes in protein expression. However, many previous studies have reported a general lack of correspondence between protein and mRNA levels under baseline conditions (Gygi et al., 1999; Gry et al., 2009; Maier et al., 2009; Ghazalpour et al., 2011). We also found minimal overlap (~20%) between our 160 most significant DE proteins and mRNA expression in a deep RNA-seq data set generated from male B6 and D2 striatum. However, there are several caveats of our analysis that may have limited detection of matched DE mRNA. First, comparisons were not made in the same individual, rather they were made across sex- and strain-matched protein and RNA-seq data sets collected at different times and in different laboratories. Second, the proteomics data set was generated following membrane enrichment while the RNA-seq data set was generated from whole striatum.

Despite some of the limitations of our analysis, there are several intriguing findings that emerged from our study. First, gene variants that perturb cognate mRNA levels appear to be more likely to also perturb expression at the cognate protein level. A similar finding was also reported by Ghazalpour et al. (2011) in that the majority (56%) of proteins that demonstrated genetic regulation of expression (pQTLs) in a genetic mouse population (the Hybrid Mouse Diversity Panel; HMDP) also demonstrated regulation of transcription (eQTL) from the same locus. However, Ghazalpour et al. (2011) were able to conduct a more global study of the genetic regulation of protein and mRNA and they reported a much higher number of eQTLs compared to pQTLs. The majority of these eQTLs failed to demonstrate conservation at the protein level.

A second interesting finding from our study is the identification of strain differences in protein expression that could contribute to behavioral differences observed between B6 and D2. For example, DE proteins expressed at a higher or lower level in D2 or B6 striatum exhibited functional enrichment for neuronal processes involved in LTP (D2 > B6) or LTD (B6 > D2) and dopaminergic (both contrasts) and glutamatergic (B6 > D2) function (Figure 3). This included higher expression of proteins involved in dopamine catabolism (COMT), regulation of dopamine levels (UBE3A, PARK7, GSTM5), and glutamate synthesis (GLUL) in B6 and dopamine synthesis (DDC) in D2. Differential expression of proteins and biological pathways related to dopamine function in the striatum is particularly relevant as these strains exhibit different and sometimes opposite behavioral and dopaminergic responses to drugs of abuse and external stimuli (Cabib et al., 2002). For example, the B6 strain may exhibit facilitation of dopamine transmission and hyperactivity under some arousing conditions, such as novel environments (Cabib et al., 2002), and is more sensitive to dopamine agonists and amphetamines relative to the D2 strain (Anisman and Cygan, 1975; Anisman et al., 1975; Phillips et al., 1994; Puglisi-Allegra and Cabib, 1997; Zocchi et al., 1998). However, under specific stressful conditions (e.g., forced swim), B6 exhibit higher passive responses, increased mesocortical dopamine metabolism and inhibition of NAC dopamine release and metabolism (Ventura et al., 2002) relative to D2. In contrast, D2 may be less sensitive to the inhibitory effects of alcohol on dopamine release (Brodie and Appel, 2000; Kapasova and Szumlinski, 2008; Yorgason et al., 2015) relative to B6.

The precise genetic and molecular mechanisms that control observed differences in striatal neurochemistry, signaling, and behavior between B6 and D2 are not known. To aid in the identification of these mechanisms, we identified candidate proteins and pathways that can be further investigated using pharmacological or genetic manipulation and forward genetic approaches. As a proof of concept, we validated higher expression in B6 relative to D2 of the primary receptor (CNR1) responsible for cannabinoid-mediated inhibition of motor behavior (Figure 5, Supplementary Figures S1, S2). We then hypothesized that the B6 and D2 strains would demonstrate differential sensitivity to the CNR1 agonist THC (10 mg/kg). In support of our hypothesis, we found that B6 males with higher baseline levels of CNR1 in striatum were more sensitive to the locomotor depressing effects of THC compared to D2 males. This is a compelling result that warrants further investigation for at least two reasons. First, there are no sequence variants in *Cnr1* segregating between B6, D2, or the BXD panel that are predicted to have an impact on mRNA or protein levels. Therefore, regulation of CNR1 and downstream signaling pathways in these strains is likely to be complex and involve multiple genes and variants. Second, CNR1-mediated modulation of motor activity by cannabinoids is equally complex and likely involves several cell-types, brain regions, and circuits (Sañudo-Peña et al., 1999; Gerdeman and Lovinger, 2001; Andersson et al., 2005; Monory et al., 2007). Although more experiments are needed to identify genetic mechanisms driving differences in the expression of CNR1 and cannabinoid sensitivity between B6 and D2, knowledge of differential protein expression and alterations in specific signaling pathways can be used to make predictions about strain response.

Although, our study was small in scale and involved the use of a small number of male animals and a single brain region, we were able to identify over 40 DE proteins likely modulated by functional variants and differences in key brain proteins and neurotransmitter pathways that are likely to explain some of the phenotypic variation between the B6 and D2 strains. We also observed the same general lack of concordance between protein and mRNA levels, although genetic variants that strongly influence mRNA levels may be more likely to also modulate protein levels. Experiments with more power to detect smaller effect changes at the protein level will help resolve some of these issues, but it is important to note how little we know about the underlying mechanisms driving divergence in mRNA and protein levels and the functional impact of DE proteins on brain signaling and behavior. Genetic variation can impact phenotypic variation at many molecular levels from transcript to protein. Systems genetics approaches combining genomic, transcriptomic, proteomic, and behavioral data collected in genetic populations, such as the HMDP or BXD recombinant inbred strains, are heuristic in understanding the functional impact of genetic variation.

## Ethics Statement

This study was carried out in accordance with the recommendations of the UTHSC Institutional Animal Care and Use Committee. The protocol was approved by the UTHSC Institutional Animal Care and Use Committee.

## Author Contributions

MM, BM, FG, and SB-G designed the experiment. CP, BM, MM, and FG performed the experiments. MM wrote the manuscript with input on methodology from CP, FG and SB-G. BJ edited the manuscript and BM and BJ provided intellectual support.

## Conflict of Interest Statement

The authors declare that the research was conducted in the absence of any commercial or financial relationships that could be construed as a potential conflict of interest.

## Footnotes

^1^amp.pharm.mssm.edu/Enrichr

^2^www.ensembl.org/biomart

^3^www.GeneNetwork.org

^4^http://visualization.ritchielab.org

## Supplementary Material

FIGURE S1 Original Western membranes. The same samples used in the proteomics analysis were used in the Western analysis. Western blot shown to left and swift stained membrane shown to right.

FIGURE S2 Additional validation of CNR1 differential expression between the B6 and D2 strains. Original Western membranes and protein quantification are shown. In Panels **(Ai)** through **(Aii)**, whole brain total protein extracts (T) were collected from a single B6 and D2 mouse. **(Ai)** Original membranes probed for CNR1 and GAPDH. Samples run in triplicate. **(Aii)** Protein quantification normalized to GAPDH levels and shown relative to expression in the D2 strain. In panels **(Bi)** through **(Biii)**, two additional B6 and D2 male mice were used to generate striatal membrane (M) and soluble cytosolic (C) membrane fractions. The resulting membrane was separated into three sections for further processing. **(Bi)** Original membrane stained with the Swift Stain kit. **(Bii)** Original membranes probed for CNR1 and GAPDH. **(Biii)** Protein quantification of membrane-enriched fractions using band intensity from the Swift stained membrane for normalization and shown relative to the D2 strain.

TABLE S1 Detected proteins.

TABLE S2 Enrichment of biological terms in differentially expressed (DE; *p* < 0.05) gene sets.

TABLE S3 DE Proteins (FDR < 10%).

TABLE S4 DE protein Regulation by cis expression quantitative trait loci (cis eQTLs).
