# Supplementary figures and images for: Comparison and Functional Genetic Analysis of Striatal Protein Expression Among Diverse Inbred Mouse Strains
Source: Front Mol Neurosci. 2019 May 24;12:128. doi: 10.3389/fnmol.2019.00128 (PMC6543464; doi:10.3389/fnmol.2019.00128)

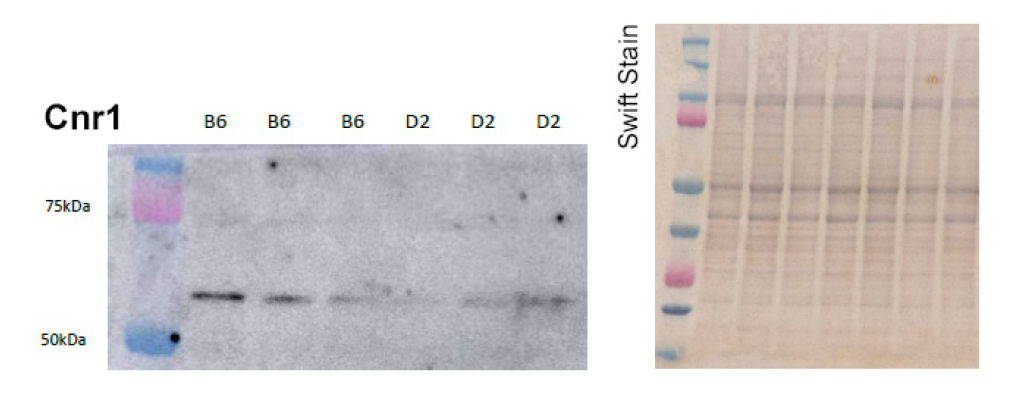

Supplement: FIGURE S1 — Original Western membranes. The same samples used in the proteomics analysis were used in the Western analysis. Western blot shown to left and swift stained membrane shown to right. [file Image_1.TIF]

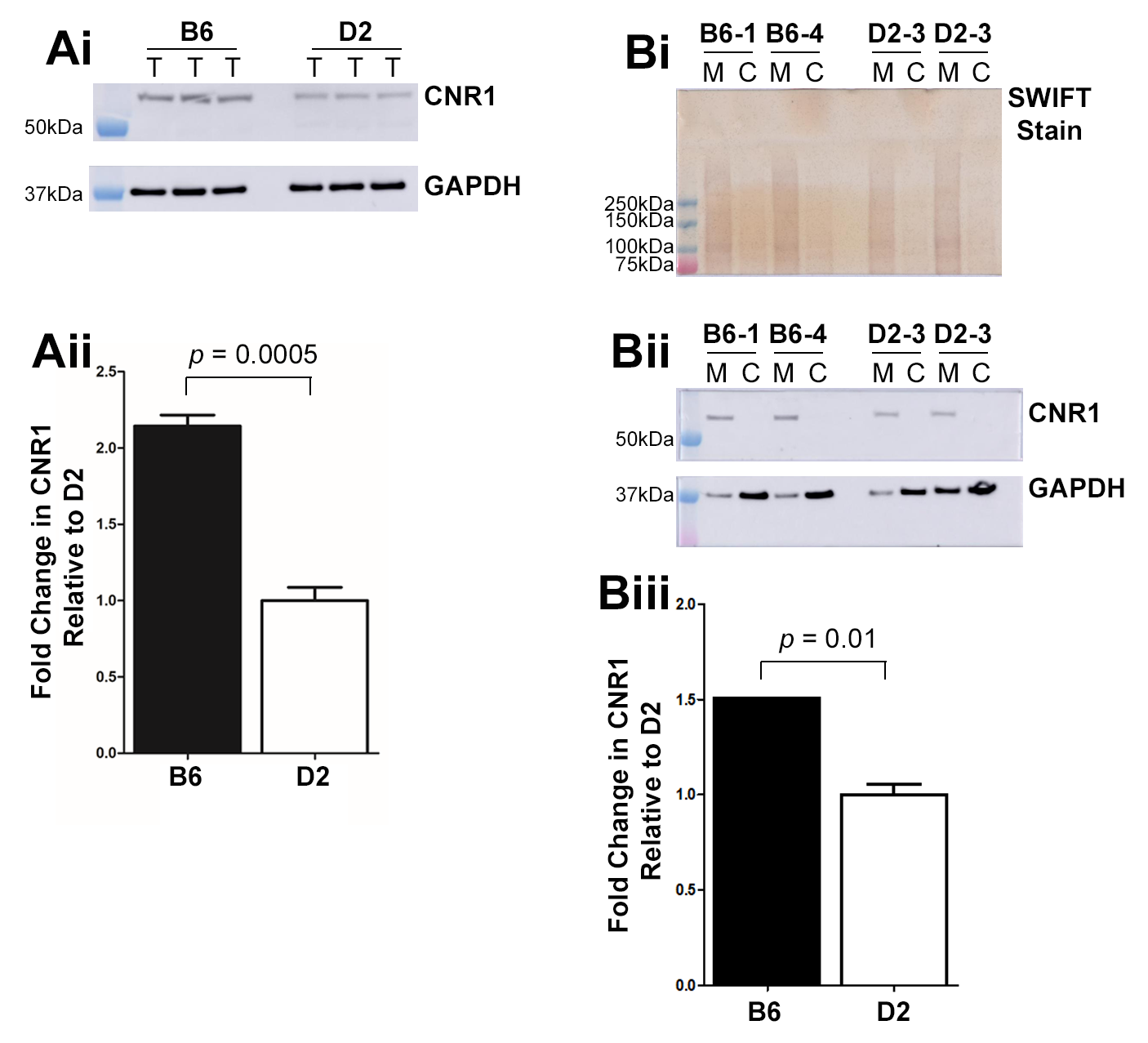

Supplement: FIGURE S2 — Additional validation of CNR1 differential expression between the B6 and D2 strains. Original Western membranes and protein quantification are shown. In Panels (Ai) through (Aii), whole brain total protein extracts (T) were collected from a single B6 and D2 mouse. (Ai) Original membranes probed for CNR1 and GAPDH. Samples run in triplicate. (Aii) Protein quantification normalized to GAPDH levels and shown relative to expression in the D2 strain. In panels (Bi) through (Biii), two additional B6 and D2 male mice were used to generate striatal membrane (M) and soluble cytosolic (C) membrane fractions. The resulting membrane was separated into three sections for further processing. (Bi) Original membrane stained with the Swift Stain kit. (Bii) Original membranes probed for CNR1 and GAPDH. (Biii) Protein quantification of membrane-enriched fractions using band intensity from the Swift stained membrane for normalization and shown relative to the D2 strain. [file Image_2.TIF]
